# Retraction: Comparison of Outcomes of Percutaneous Nephrolithotomy (PCNL) Between Adults and Pediatrics Population: A Single-Center Retrospective Study

**DOI:** 10.7759/cureus.r54

**Published:** 2022-03-17

**Authors:** Muhammad Rizwan Umer, Marina Basta, Sandrine Kakieu Djossi, Amin Tafti, Musharaf Khan, Maria Binte Sarfraz, Sabeen Sharif Khan, Jobby John, Khizer Shamim

**Affiliations:** 1 Medical School, District Head Quarter Hospital, Nankana Sahib, PAK; 2 Medical School, St. George's University, True Blue, GRD; 3 Medical School, California Institute of Behavioral Neurosciences and Psychology, Fairfield, USA; 4 Medical School, Virginia Commonwealth University, Richmond, USA; 5 Neurosurgery, Combined Military Hospital, Rawalpindi, PAK; 6 Physiology, Army Medical College, National University of Medical Sciences, Rawalpindi, PAK; 7 Medicine, Aga Khan University Hospital, Karachi, PAK; 8 Internal Medicine, Dr. Somervell Memorial CSI Medical College, Thiruvananthapuram, IND; 9 Medical School, Ziauddin University Hospital, Karachi, PAK

This article has been retracted due to the unknown origin of the data, lack of verified IRB approval, and purchased authorships. While not listed as an author, it was discovered that Rahil Barkat wrote and coordinated the submission of this article. Mr. Barkat was involved in data theft and misuse in two recently published Cureus articles, which have since been retracted.

As the origin of this article’s data and verified IRB approval cannot be confirmed, we have made the decision to retract this article. Cureus has confirmed that the co-authors were asked by Mr. Barkat to proofread the article and provide payment in exchange for authorship. (Proofreading is an insufficient contribution to warrant authorship as defined by ICMJE.) These payments were made in the guise of “editing fees” but greatly exceed any editing fees paid to Cureus. While these authors may have been defrauded by Mr. Barkat, they remain complicit due to their lack of honest contributions to the article.

